# CPT1C promotes human mesenchymal stem cells survival under glucose deprivation through the modulation of autophagy

**DOI:** 10.1038/s41598-018-25485-7

**Published:** 2018-05-03

**Authors:** Xavier Roa-Mansergas, Rut Fadó, Maher Atari, Joan F. Mir, Helena Muley, Dolors Serra, Núria Casals

**Affiliations:** 1 Basic Sciences Department, Faculty of Medicine and Health Sciences, Universitat Internacional de Catalunya (UIC), 08195 Sant Cugat del Vallès, Spain; 2 Regenerative Medicine Institute, Universitat Internacional de Catalunya, 08195 Sant Cugat del Vallès, Spain; 3 Department of Biochemistry and Physiology, Faculty of Pharmacy, Institut de Biomedicina de la Universitat de Barcelona (IBUB), Universitat de Barcelona, 08028 Barcelona, Spain; 4 Centro de Investigación Biomédica en Red de Fisiopatología de la Obesidad y la Nutrición (CIBEROBN), Instituto de Salud Carlos III, Madrid, Spain

## Abstract

Human mesenchymal stem cells (hMSCs) are widely used in regenerative medicine. In some applications, they must survive under low nutrient conditions engendered by avascularity. Strategies to improve hMSCs survival may be of high relevance in tissue engineering. Carnitine palmitoyltransferase 1 C (CPT1C) is a pseudoenzyme exclusively expressed in neurons and cancer cells. In the present study, we show that CPT1C is also expressed in hMSCs and protects them against glucose starvation, glycolysis inhibition, and oxygen/glucose deprivation. CPT1C overexpression in hMSCs did not increase fatty acid oxidation capacity, indicating that the role of CPT1C in these cells is different from that described in tumor cells. The increased survival of CPT1C-overexpressing hMSCs observed during glucose deficiency was found to be the result of autophagy enhancement, leading to a greater number of lipid droplets and increased intracellular ATP levels. In fact, inhibition of autophagy or lipolysis was observed to completely block the protective effects of CPT1C. Our results indicate that CPT1C-mediated autophagy enhancement in glucose deprivation conditions allows a greater availability of lipids to be used as fuel substrate for ATP generation, revealing a new role of CPT1C in stem cell adaptation to low nutrient environments.

## Introduction

Mesenchymal stem cells (MSCs) are multipotent stem cells that can differentiate into various tissues of mesenchymal origin, including adipocytes, chondrocytes, osteocytes or myocytes, and even transdifferentiate into other embryonic lineage tissues, such as neurons or corneal cells^1–3^. They are attractive candidates for cell therapies and regenerative medicine because of their minimally invasive isolation procedure, low immunogenicity, low tumorigenic potential, and prevalent homing to injured tissues^4^. MSCs are highly glycolytic^5^, with glucose deficiency being one of the challenges that MSCs have to face during tissue regeneration since injury may disrupt the blood supply bringing nutrients to the damaged area^6^. Therefore, determining how to improve the survival of MSCs in low-nutrient environments may be extremely useful.

Carnitine palmitoyltransferase 1 (CPT1) is a family of enzymes that catalyze the exchange of long chain acyl groups between carnitine and CoA to facilitate the transport of long-chain fatty acids from the cytoplasm to the lumen of the mitochondria for β-oxidation^7^. Unlike mitochondrial-resident isoforms CPT1A and CPT1B, CPT1C is located in the endoplasmic reticulum (ER) and exhibits residual catalytic activity *in vitro* with palmitoyl-CoA^8–10^. Moreover, several acyl-CoAs of different lengths, saturation grade or ramification were tested but CPT1C showed no carnitine acyltransferase activity with any of them. However, CPT1C maintains the ability to bind the physiological inhibitor of CPT1 enzymes, malonyl-CoA^9,10^, the intracellular levels of which fluctuate depending on nutrient availability^11^. In fact, CPT1C has been proposed to be a sensor of malonyl-CoA levels in cells^12^. Interestingly, CPT1C is only expressed in neurons and tumor cells in mammals^9,13^. Studies with knock-out (KO) mice have demonstrated that neuronal CPT1C is involved in spatial learning^14–16^, motor function^17,18^, and the hypothalamic control of food intake and energy expenditure^19–21^. Additionally, CPT1C allows tumor cells to survive in hypoglycemic and hypoxic conditions^13,22^, while CPT1C silencing leads to delayed tumor development^22^. Moreover, CPT1C is 1 of the 5 gene signature that is strongly associated with the epithelial-mesenchymal program across multiple cancers^23^.

In the present study we show for the first time that CPT1C is expressed in human adult mesenchymal stem cells (hMSCs) and located in ER-mitochondria contact sites, and that CPT1C promotes cell survival under glucose deficiency conditions through the enhancement of the autophagic flux and lipid droplet synthesis. This work unravels a new role of CPT1C different from the previous ones described in tumor cells or neurons, and identifies CPT1C as a possible target in strategies aimed to improve the survival of hMSCs in regenerative medicine.

## Results

### CPT1C is expressed in hMSCs

**Figure 1:**
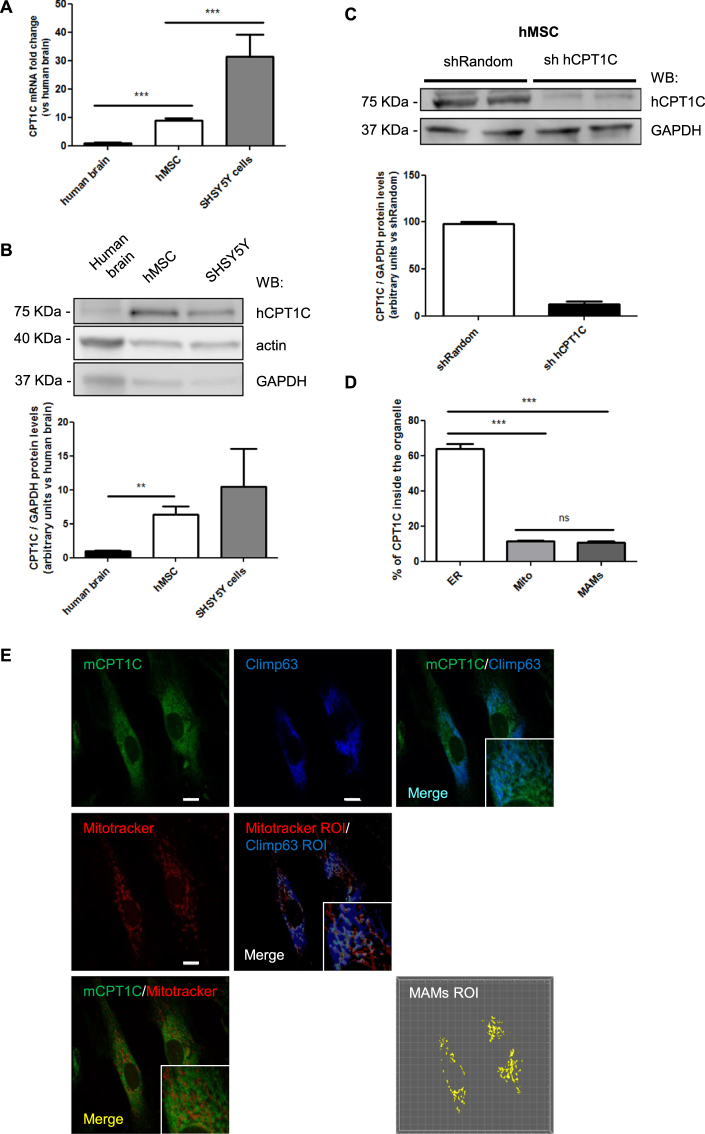
CPT1C is expressed in adult hMSCs. (**A**) CPT1C mRNA levels were determined by real-time PCR in human brain, hMSCs and SH-SY5H cell line. Actin was used as a housekeeping gene. Results are shown as mean ± SEM of sextuplet. (**B**) Endogenous CPT1C was analyzed by Western Blot in hMSCs, human brain cells, and the SH-SY5Y human neuroblastoma cell line. GAPDH was used as a loading control. n = 3–6. (**C**) hMSCs were infected with a lentivirus expressing a random sequence (Random) or a silencing sequence for human CPT1C (sh hCPT1C). Transduced cells were selected by FACS. Expression of CPT1C analyzed by Western blot showed an 80% of reduction in silenced cells (sh hCPT1C). GAPDH was used as a loading control. Graph shows the mean ± SEM of 3 independent experiments performed in duplicate. (**D**,**E**) hMSCs were infected with a lentivirus to drive the expression of mouse CPT1C (pWPI-mCPT1C-IRES-GFP). Mitotracker Orange was used to stain mitochondria, while mCPT1C and Climp63, the latter used as an ER marker, were detected by immunocytochemistry. EV-hMSCs were used as a negative control for CPT1C immunocytochemistry (see the images in Supplemental Fig. 2).The graph shows the percentage of CPT1C inside the ER, in the mitochondria, or on the surface (region of interest, ROI) defined by the colocalization of the ER with the mitochondria (MAMs). Values shown in D are expressed as mean ± SEM of 3 independent experiments performed in duplicate (10 randomly selected cells per coverslips were analyzed). Scale bar 10 µm. *p < 0.05; ***p < 0.001.

To study whether CPT1C is expressed in human adult stem cells, we used hMSCs derived from dental pulp kindly provide by the Regenerative Medicine Research Institute (RMI), at UIC^24,25^. hMSCs were used at 6–9 passages in all the experiments. We analyzed CPT1C mRNA levels in hMSCs and compared them to those present in the human brain. The neuroblastoma cell line SH-SY5Y was used as a positive control. Figure 1A shows how CPT1C mRNA levels in hMSCs were found to be 9 times higher than in the human brain (1.00 ± 0.06 for brain vs 8.92 ± 0.85 for hMSCs). At the protein level, we observed a 75 kDa band more intense in hMSCs than in human brain (Fig. 1B; 1.00 ± 0.10 for brain vs 6.34 ± 1.22 for hMSCs) and similar to the SHSY5Y cells. Taking into account that CPT1C is exclusively present in neurons and not in glial cells^8^, and that neurons represent about 10% of brain cells, we can say that the CPT1 expression in hMSC is similar to the one in neurons.

Given that the human CPT1C (hCPT1C) molecular mass was predicted to be 91 kDa, we decided to validate the specificity of the CPT1C commercial antibody. We overexpressed hCPT1C in HEK293T cells and found an intense 75-kDa band (Supplemental Fig. 1A) that was reduced by CPT1C-shRNA co-transfection (Supplemental Fig. 1B). We then silenced the endogenous CPT1C in hMSCs using lentivirus (sh-hCPT1C) and observed that the intensity of the 75-kDa band was clearly reduced (about 80% compared to control cells; Fig. 1C), confirming that hCPT1C migrates with an electrophoretic mobility of 75 kDa, as previously described for mouse and rat CPT1C^8,9^. With these findings, we demonstrate that CPT1C is expressed not only in neurons and tumor cells, but also in hMSCs.

We then analyzed whether CPT1C was present in the ER of hMSCs as it had been described in neurons^8^. Since CPT1C commercial antibodies did not work to detect endogenous CPT1C by immunohistochemistry, we overexpressed mouse CPT1C in hMSCs using lentivirus (pWPI-mCPT1C-IRES-GFP). CPT1C was stained with mouse anti-CPT1C, ER with anti-Climp63 and mitochondria with Mitotracker (see Supplemental Fig. 2A–C for antibody specificity controls). ER-mitochondria contact sites (also known as mitochondria-associated membranes, or MAMs) were identified using colocalization of Climp63 with Mitotracker. Our results show that CPT1C was mainly located in the ER (Fig. 1D,E) but also in a small proportion (approx. 15%) in the mitochondria. Similar results were observed overexpressing hCPT1C tagged with turquoise fluorescent protein in HEK293T cells and using calnexin as ER marker (Supplemental Fig. 3A,B). The fact that CPT1C is present in the ER-mitochondria contact sites with the same percentage as in mitochondria suggests that CPT1C is located on the ER side of these contacts.

### CPT1C protects hMSCs against cellular damage induced by glucose depletion, 2-deoxyglucose (2-DG) treatment, and oxygen/glucose depletion (OGD)

**Figure 2:**
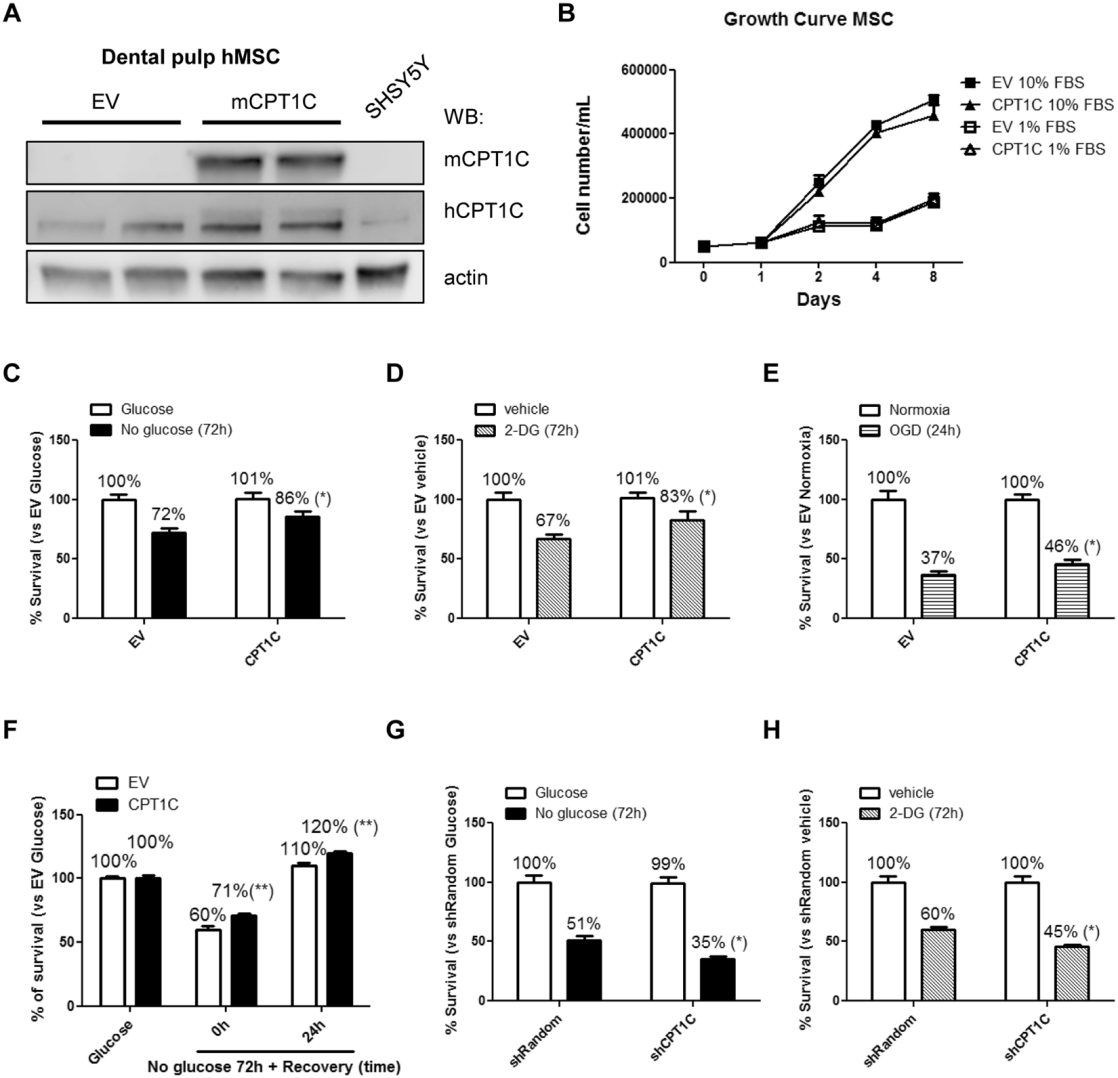
CPT1C overexpression increases survival of hMSCs against cellular damage induced by glucose depletion or 2-deoxyglucose treatment. (**A**) Dental pulp MSCs were transduced with lentivirus containing the empty vector (EV) or the vector codifying for mCPT1C and subsequently separated by FACS. Endogenous human CPT1C and overexpressed mouse isoform were detected by western blot using antibodies specific to mouse CPT1c (mCPT1C) or commercial antibodies (SIGMA SAB2501194) that recognize both human and mouse CPT1C (hCPT1C). β-actin was used as a loading control. (**B**) Growth curve during 8 days of EV (squares) or CPT1C (triangles) –hMSCs cultured with 10% FBS or 1% FBS. (**C**–**H**) Cell survival analysis were performed in media with 1% FBS. EV or CPT1C-hMSCs were cultured in glucose-deprived media for 72 h (**C**), treated with 50 mM 2-DG for 72 h (D), OGD for 24 h (**E**). In (**F**), cells were glucose starved for 72 h and then returned to a medium with glucose for additional 24 h. Random or CPT1C-silenced cells were cultured in glucose depleted media (**G**) or treated with 2-DG (**H**) for 72 h. All results are shown as mean ± SEM of 3 independent experiments performed in sextuplet. *p < 0.05.

We looked at whether CPT1C overexpression would confer protection to hMSCs under different situations of metabolic stress, as previously described in tumor cells. To that end, we permanently overexpressed mouse CPT1C in hMSCs using a lentiviral vector (pWPI-mCPT1C-IRES-GFP) and carried out posterior FACS selection of the transduced cells (Fig. 2A), which were named CPT1C-hMSCs. Control cells were those transduced with the empty vector (EV-hMSCs). Given that CPT1C human antibody (Sigma SAB2501194) was able to recognize mouse overexpressed isoform (see Methods section), we used it to analyze the magnitude of the increase in CPT1C overexpression. Figure 2A shows that CPT1C expression was increased by 44% in CPT1C-MSCs compared to control cells (EV-MSCs). Additionally, we confirmed that the transduced cells maintained their multipotency by differentiating them to adipocytes and osteoblasts (Supplemental Fig. 4).

We tested whether CPT1C overexpression influenced cell proliferation. As shown in Fig. 2B, no significant differences in cell number were observed between CPT1C-hMSCs and EV-hMSCs at different concentrations of serum and on different days. The following experiments were carried out at the concentration of 1% serum to minimize proliferation.

To test whether CPT1C overexpression promoted cell survival under starvation, cells were glucose deprived or submitted to treatment with 2-DG, a glycolysis inhibitor, for 72 hours. In both experiments CPT1C-hMSCs survived approximately 20% to 25% more than in control cells (glucose deprivation: 72.07% ± 3.39 for EV vs 85.56% ± 4.18 for CPT1C; 2-DG: 66.76% ± 4.07 for EV vs 82.75% ± 7.46 for CPT1C; Fig. 2C,D). Similar results were observed assessing cellular mortality by the trypan blue exclusion method in glucose depleted conditions (Supplemental Fig. 5A). Furthermore, glucose starvation induced a long term increase in CPT1C endogenous protein levels (48 hours; Supplemental Fig. 5B). We then tested CPT1C effects under oxygen-glucose deprived conditions (OGD). Cells were maintained in media without glucose and at 0.6% O_2_ for 24 hours. CPT1C over-expression increased cell survival by 24% (OGD: 37.13% ± 3.08 for EV vs 46.22% ± 3.24 for CPT1C; Fig. 2E). Additionally, we performed a glucose recovery experiment after starvation and we observed comparable protective effects of CPT1C (no glucose 72 h + recovery 24 h: 110.04% ± 2.10 for EV vs 119.56% ± 1.72 for CPT1C; Fig. 2F). Finally, we tested other metabolic stressors such as H_2_O_2_ (an oxidative stressor), thapsigargin (a disruptor of free intracellular Ca2+ levels), and sodium palmitate (a lipotoxicity inductor). In none of these cases CPT1C overexpression was able to attenuate cell mortality induced by those treatments (Supplemental Fig. 5C–E).

To confirm the protective role of CPT1C in hMSCs from other source, we overexpressed CPT1C in bone marrow-derived hMSCs (BM-hMSCs; Supplemental Fig. 5F). We observed that under glucose depletion or 2-DG treatment, CPT1C overexpression also exerted protective effects on BM-hMSCs (glucose deprivation: 54.00% ± 2.81 for EV vs 64.12% ± 2.72 for CPT1C and 2-DG: 60.42% ± 1.67 for EV vs 45.51% ± 1.49 for CPT1C; Supplemental Fig. 5G,H).

Next, we tested whether CPT1C silencing in hMSCs would decrease cell survival under glucose deprivation or 2-DG treatment. As shown in Fig. 2G,H, CPT1C silencing increased mortality by 30% in both conditions (glucose deprivation survival: 51.36% ± 3.84 for EV vs 35.07% ± 2.41 for CPT1C; and 2-DG survival: 60.42% ± 1.67 for EV vs 45.51% ± 1.49 for CPT1C). Our results demonstrate that CPT1C confers long lasting protection to hMSCs when glucose availability or glucose catabolism is hindered.

### CPT1C overexpression blocks the starvation-induced decrease of intracellular ATP levels

**Figure 3:**
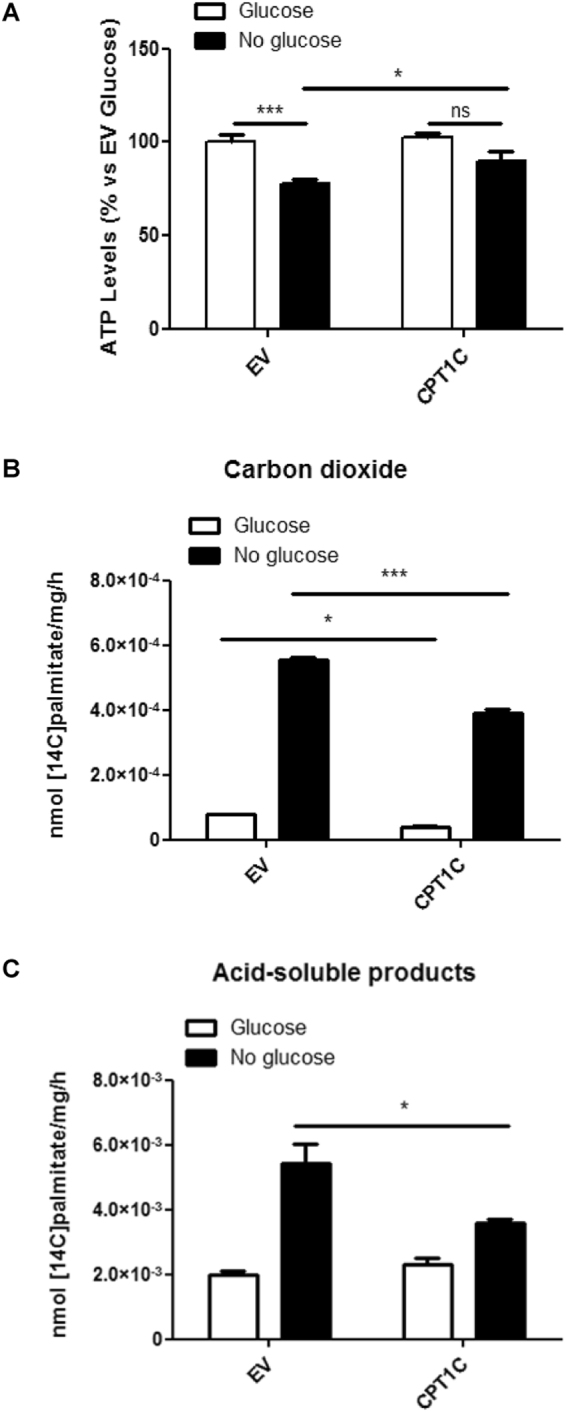
CPT1C overexpression in hMSCs leads to higher ATP production in glucose deprived media even though maximal FAO capacity is not increased compared to control cells. (**A**) ATP levels were measured in EV or CPT1C-hMSCs in the presence (white bars) or the absence (36 h) (black bars) of glucose. Results are shown as mean ± SEM of 3 independent experiments performed in triplicate. (**B**,**C**) FAO was determined in EV or CPT1C-hMSCs in the presence (5 mM) or absence of glucose (24 h). nmol (^14^C) palmitate/mg/h converted to carbon dioxide (**B**) and ASP (**C**) were measured. Results are shown as mean ± SEM of triplicate. *p < 0.05; ***p < 0.001.

Since glucose is the main fuel substrate for ATP production in stem cells^26^, we decided to measure ATP levels in cell extracts under glucose deprivation. Our results showed that glucose starvation triggered a significant reduction in ATP levels in control cells, but not in CPT1C-hMSCs (Fig. 3A), indicating that CPT1C plays a role in the maintenance of ATP levels during starvation.

Although CPT1C has residual catalytic activity (2–4) and is mainly located in the ER of hMSCs, we chose to investigate whether CPT1C overexpression enhanced maximal mitochondrial FAO in hMSCs, as previously described in human tumor cells^13^. ^14^C-palmitate oxidation was measured as CO_2_ (Fig. 3B) or ASP (acid soluble products, essentially ketone bodies; Fig. 3C) formation in the presence (5 mM) or in the absence of glucose. Since saturant concentrations of ^14^C-palmitate were used, the maximal FAO capacity of mitochondria was measured. Results showed that FAO was highly increased when media was deprived of glucose for 24 hours in both EV-hMSCs and CPT1C-hMSCs; however, CPT1C overexpression attenuated ^14^C-palmitate oxidation when compared to the control cells. We can conclude that CPT1C does not increase hMSCs survival through the enhancement of maximal FAO capacity of the cells. These results are coherent with previous published data that demonstrates that CPT1C overexpression in COS-1 or PC12 cells does not increase FAO^8,19^.

### Autophagy activation is required for CPT1C-enhanced survival of hMSCs

Since CPT1C confers protection to hMSCs in glucose-depleted conditions and was found not to be mediated by the enhancement of FAO capacity, we looked at autophagy activation as a means of obtaining energy from the degradation of bulk cytosol and organelles.

**Figure 4:**
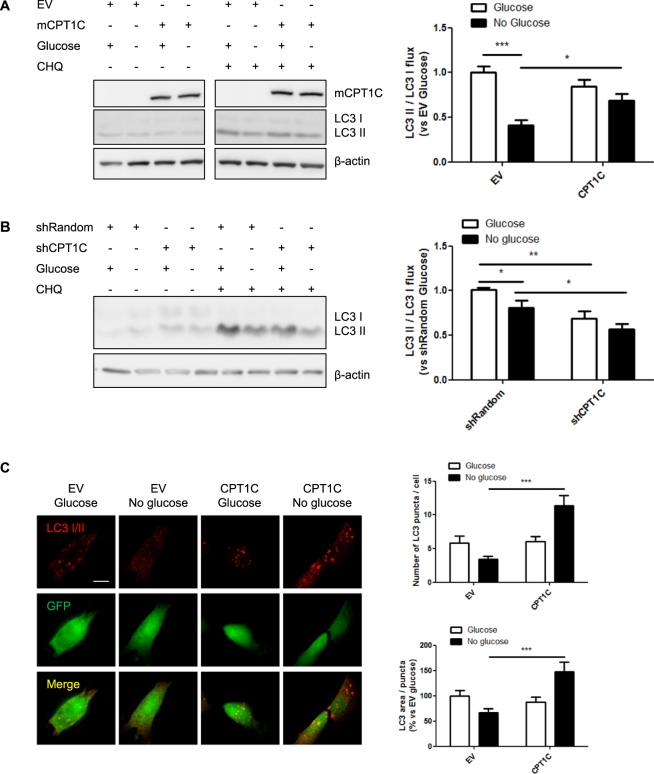
CPT1C overexpression promotes autophagy under glucose-depleted conditions. (**A**,**B**) Quantification of the LC3II/LC3I flux in EV and CPT1C-hMSCs (**A**) or shRandom and shCPT1C-hMSCs (B) in the presence or the absence (24 h) of glucose. In some of the wells, 50 µM chloroquine was added 1 h before cell recollection. LC3 I and LC3 II were analyzed by Western Blot. GFP was used as a transduction control and β-actin as a loading control. Blots displayed in A were cropped from different parts of the same gel (see full-length blot in Supplemental Fig. 6A). Results are shown as mean ± SEM of 3 independent experiments performed in duplicate. (**C**) LC3 was detected by immunocytochemistry in EV- or CPT1C-hMSCs 24 h after glucose depletion. Twenty randomly selected cells per condition were analyzed in 5 independent experiments performed in duplicate. The graphs show the number of LC3 puncta per cell (above) and the LC3 area per puncta (down) and are expressed as mean ± SEM. Scale bar 20 µm. *p < 0.05; ***p < 0.001.

We first analyzed the conversion of the cytosolic form of LC3 (LC3-I) to the lipidated one (LC3-II) by measuring the LC3-II/LC3-I flux in the presence or absence of chloroquine (CHQ), an inhibitor of the fusion of autophagosome with lysosome. When the autophagy flux was blocked with CHQ, a reduction in autophagosome accumulation was observed in control cells (EV-hMSCs) during glucose deficiency compared to complete media conditions (EV: 1.00 ± 0.09 for glucose vs 0.34 ± 0.11 for non-glucose; Fig. 4A), as previously described in other cell types^27^. However, this reduction was not observed in CPT1C-hMSCs, the LC3 II/LC3 flux being similar to that of the non-deprived cells (CPT1C: 0.95 ± 0.14 for glucose vs 0.82 ± 0.21 for non-glucose; Fig. 4A), indicating that CPT1C overexpression prevents the decrease of autophagic flux induced by glucose deprivation. We then analyzed the autophagic flux in CPT1C-silenced cells and found it to be reduced compared to control cells (Fig. 4B), both in control (1.00 ± 0.02 for EV vs 0.69 ± 0.08 for CPT1C) and glucose depleted conditions (0.81 ± 0.08 for EV vs 0.57 ± 0.06 for CPT1C), confirming the role of CPT1C in the regulation of the autophagy flux by glucose starvation.

**Figure 5:**
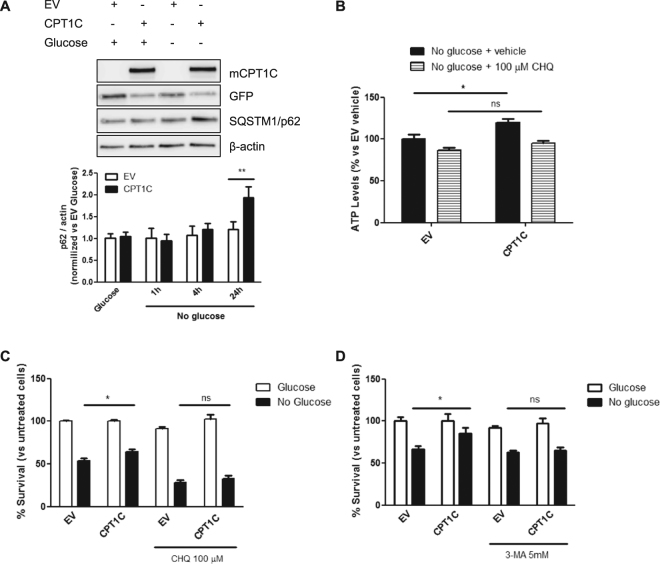
Autophagy inhibition blocks CPT1C protective effects against glucose starvation. (**A**) p62 protein levels were analyzed by immunoblotting in EV or CPT1C-hMSCs at the indicated times after glucose depletion. (**B**) ATP production was evaluated after 36 h of glucose starvation in the presence (last 24 h) or absence of 100 µM chloroquine. Results are shown as mean ± SEM of 2 independent experiments performed in triplicate. (**C**,**D**) Cellular viability was measured in EV or CPT1C-hMSCs in the presence or absence of glucose for 72 h with or without the addition of chloroquine (in **C**) or 3-MA (in **D**) in the last 24 h. Results are shown as mean ± SEM of 3 or 4 independent experiments performed in sextuplet. *p < 0.05.

We next analyzed LC3 puncta by immunohistochemistry and found that CPT1C-hMSCs showed a higher LC3 area per puncta and number of puncta per cell during fasting conditions when compared to control cells (Fig. 4C). p62 protein levels increased after 24 hours of glucose deprivation (Fig. 5A), while mRNA levels did not change (Supplemental Fig. 6B). This positive correlation between p62 protein levels and autophagic activity had been described in mouse embryonic stem cells (MEFs) and in a HepG2 cell line, where prolonged periods of starvation (more than 4 h) led to increased levels of p62 and higher autophagosome number^28^.

To confirm that autophagy activation is necessary for the protective effects of CPT1C, we blocked autophagy with chloroquine and analyzed ATP levels and cell survival. Our results showed that CPT1C-hMSCs treated with chloroquine were unable to produce more ATP (Fig. 5B) or survive more than the control cells under glucose-deprived conditions (28.44 ± 2.56 for EV CHQ vs 32.90 ± 3.29 for CPT1C CHQ; Fig. 5C), indicating that the enhancement of autophagy is required for the protective effects of CPT1C during glucose deprivation. The same results were obtained when autophagy was blocked with 3-methyladenine (3-MA), an inhibitor of autophagosome formation through the inhibition of type III Phosphatidylinositol 3-kinases (Fig. 5D). Based on these findings, we conclude that CPT1C overexpression confers protection under glucose deprivation through the enhancement of autophagy.

### Lipolysis of autophagy-derived lipid droplets (LDs) is necessary for CPT1C-induced protection

**Figure 6:**
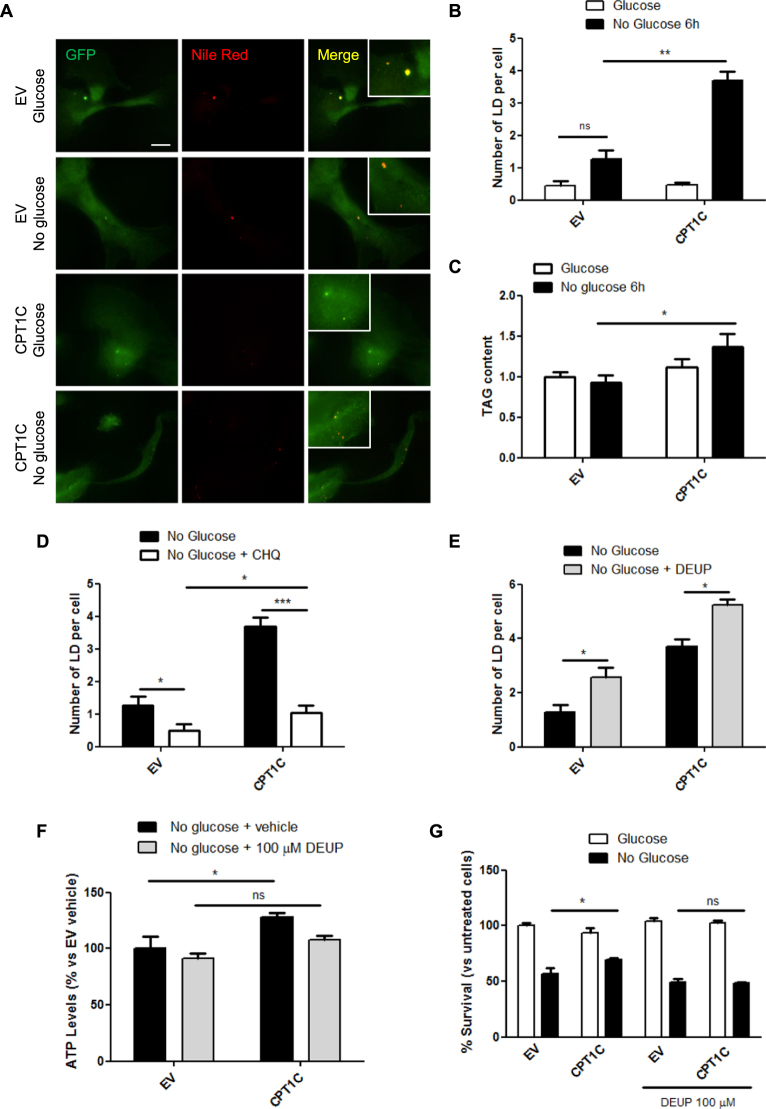
An increase in LD synthesis is essential to the CPT1C-mediated protector effect under glucose starvation. (**A**) Analysis of LD number and area in EV or CPT1C-hMSCs cultured with or without glucose for 6 h. Cells were stained with Hoechst (blue) or Nile Red (red). Representative images are shown in A. The number of LDs per cell (**B**) are shown as the mean ± SEM of 3 independent experiments performed in triplicate. (**C**) Triglyceride content was determined 6 h after glucose starvation. Relative values are the mean ± SEM of 4 independent experiments performed in triplicate. (**D**–**E**) EV or CPT1C-hMSCs were cultured in the absence of glucose (6 h) in the presence or absence of 100 µM CHQ (D) or 100 µM DEUP (**E**). Results are shown as mean ± SEM of 3 independent experiments performed in triplicate. (**F**) ATP levels were measured in EV or CPT1C-hMSCs under glucose deprivation (36 h) in the presence (last 24 h) or absence of 100 µM DEUP. Results are shown as the mean ± SEM of 2 independent experiments performed in triplicate. (**G**) Cell viability was measured in EV or CPT1C-hMSCs in the presence or absence of glucose (72 h) with or without the addition of 100 µM DEUP in the last 24 h. Cell survival results are shown as mean ± SEM of 4 independent experiments performed in sextuplet. *p < 0.05; **p < 0.01; ***p < 0.001.

Given that the stimulation of autophagy results in LDs accumulation^29^, we decided to analyze whether LD synthesis was essential for the CPT1C-mediated protection of hMSCs. As expected, LDs accumulated in the cytosol of glucose-deprived cells. However, this accumulation was highly increased in CPT1C-hMSCs (Fig. 6A,B, Supplemental Fig. 6D). Interestingly, the number of LDs increased while the LD area did not change (Supplemental Fig. 6C). Moreover, this higher number of LD in CPT1C-hMSCs after glucose deprivation correlated with a significant increase in total triglyceride (TAG) content compared to control cells (Fig. 6C). These results are coherent with previous published data that demonstrates that CPT1 overexpression in COS7 cells and in cultured neurons highly increases LD number^18^. Next, we blocked the autophagic cycle with chloroquine to determine whether the increase in LDs was arrested. Figure 6D shows how LD number clearly diminished due to chloroquine treatment, suggesting that most LDs synthesized in glucose-deprived conditions were derived from the autophagic flux. In addition, we analyzed LDs in CPT1C-silenced hMSCs and found that the number of LDs per cell was reduced compared to control cells in both glucose containing and glucose depleted media (Supplemental Fig. 7A,D).

It has been described that lipolysis is a crucial step in fatty acid release from LDs during starvation, which are then transferred to mitochondria for their β-oxidation^30^. Hence, we then blocked triacylglycerol lipolysis by inhibiting the ATGL enzyme with diethylumbelliferyl phosphate (DEUP) to analyze whether CPT1C-mediated cell survival was dependent on LD lipolysis. DEUP treatment resulted in LD accumulation (Fig. 6E) verifying that LDs cannot be used as fuel in these conditions. As a consequence, the increase in ATP levels (Fig. 6F) and survival (Fig. 6G) induced by CPT1C overexpression in hMSCs grown in glucose-depleted media was completely blocked by DEUP treatment. As a whole, these results confirm that CPT1C promotes autophagy resulting in a higher accumulation of lipid stores and that LD lipolysis is required for CPT1C-mediated increase of ATP and survival effects.

**Figure 7:**
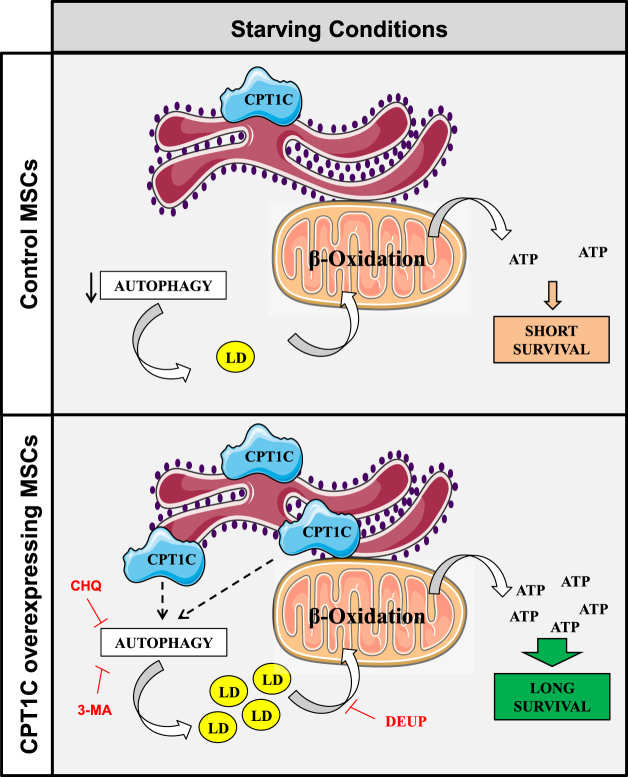
The suggested mechanism by which CPT1C increases cell survival in hMSCs under glucose starvation. hMSCs show reduced autophagic flux under glucose starvation. CPT1C overexpression increases autophagy and LD number in glucose-deficient conditions, which results in increased fatty acid availability for mitochondrial β-oxidation, and consequently, higher levels of ATP and longer cell survival compared to control cells.

In summary, results show that complete glucose depletion from media causes a decrease in the autophagic flux in hMSCs but not in those cells that over-express CPT1C. The maintenance of autophagy in CPT1C-hMSCs leads to a much higher increase of LDs during glucose deprivation than in control cells. This increased availability of lipids allows the cells to use them as fuel substrate and generate higher quantities of ATP for longer periods of time, which favors a more extended cell survival (see Fig. 7 for a scheme).

## Discussion

This study describes the expression of CPT1C in hMSCs and demonstrates its protective role against different glucose-deprived conditions such as glucose deficiency, glycolysis inhibition, and OGD, but not against other cellular stresses, such as H_2_O_2_, thapsigargin, and palmitate treatment. Thus, CPT1C plays a role in the activation of survival pathways when glucose availability is compromised, resulting in improved energy management.

A relevant finding of this work is that CPT1C promotes hMSCs survival by the enhancement of autophagy, and not by the increase of FAO capacity, as previously described in cancer cells^13^. The fact that CPT1C does not increase maximal FAO in hMSCs is coherent with the fact that CPT1C is located in the ER instead of mitochondria and has residual catalytic activity^8^. Moreover, in different cell types, such as COS-1, and PC12 cells, and brain slices CPT1C was not able to increase FAO^8,19^. The findings of the present work show that the metabolic advantage acquired by CPT1C-hMSCs was the enhancement of autophagy during glucose deprivation, as a key mechanism to providing higher quantity of fuel substrates for the generation of ATP and the maintenance of cell survival. Autophagy plays a key role during nutrient scarcity because it provides the cells with glucose, fatty acids and aminoacids derived from the digestion of organelles and/or bulk cytosol. Fatty acids are one of the main metabolites produced as a result of autophagy^31,32^. In fact, LD number in CPT1C-overexpressing cells was highly increased 6 hours after glucose deprivation compared to control cells. These results are coherent with previous published data that demonstrates that CPT1 overexpression in COS7 cells and in cultured neurons highly increases LD number when treated with oleate^18^.

We postulate that the lipolysis of LDs provides mitochondria with the proper fuel substrate (acyl-CoAs) to generate ATP through β-oxidation, as previously described^30,33^. Even though FAO maximal capacity is slightly lower in CPT1C-hMSCs than in control cells, they are able to oxidize more fatty acids generating higher quantities of ATP and allowing the cells to survive for longer periods of time. By contrast, control cells, are more vulnerable to glucose starvation and more prone to die because of autophagy attenuation and the consequent lack of fuel substrate. It would be of great interest to study whether CPT1C also confers protection under glucose-depleted conditions in neurons, as it does in adult stem cells.

It is well known that the energy sensor AMP-dependent kinase (AMPK) phosphorylates and inactivates acetyl-CoA carboxylase (ACC), resulting in a decrease of cellular malonyl-CoA levels^34^. Given that CPT1C can bind malonyl-CoA^9,10^, the physiological inhibitor of CPT1 enzymes, we hypothesize that CPT1C becomes functional in situations of nutrient scarcity when AMPK is activated and malonyl-CoA levels decrease, as occurs in cells grown in glucose-depleted media. Although the molecular mechanism by which CPT1C enhances autophagy is unknown, it is interesting to note that CPT1C is localized in the ER-mitochondria contact sites in hMSCs (Fig. 1E), a defined site for autophagosome formation during starvation^35^. This suggests that CPT1C could be involved in the transport of lipids between the 2 organelles, a necessary function for the growth of the isolation membrane during autophagy initiation. As CPT1C shows residual catalytic activity, we hypothesize that it regulates the function of other proteins involved in the autophagy process. In fact, it has recently been described that the phosphatase Sac1 is an strong interactor of CPT1C^36^. Sac1 regulates phosphatidylinositol 3-phosphate (PI3P) and 4-phosphate (PI4P) levels in cell membranes^37^, which are key signaling lipids for autophagy initiation and autophagosome maturation into autolysosomes, respectively^38,39^. Future experiments are needed to elucidate the role of CPT1C-Sac1 interaction in the regulation of the autophagic flux.

Autophagy plays a relevant role in the homeostatic control and maintenance of stem cell self-renewal. Constitutive autophagy levels in hMSCs are high and dramatically decrease when these cells differentiate^31,32,40,41^. We postulate that the maintenance of the autophagic flux in CPT1C-overexpressing cells not only promotes cell survival in conditions of glucose deficiency, but also promotes the maintenance of cell *stemness* when nutrient availability is compromised.

In summary, we report for the first time that CPT1C is expressed in adult hMSCs and that it promotes cell survival under glucose deficiency by increasing autophagy and lipid availability as a fuel substrate for the generation of ATP. The identification of proteins that improve hMSC survival under stressful conditions may be useful in regenerative medicine. hMSCs used in bone and cartilage tissue engineering must survive and produce extracellular matrix under low nutrient conditions engendered by avascularity, the central regions of implanted 3D constructs being one example^6^. A strategy involving the preselection of subpopulations that highly express CPT1C might have the potential to improve clinical outcomes based on the higher resilience of these cells to metabolic challenges.

## Methods

### hMSC isolation and culture

MSCs obtained from human dental pulp were kindly provided by the Regenerative Medicine Research Institute (RMI), at UIC^24^. Healthy third molars were extracted for orthodontic and prophylactic reasons and a sample was taken for MSC isolation from 1 randomly selected adult patient, who had given informed consent. All experiments were approved and performed in accordance with the guidelines on human stem cell research issued by the Committee on Bioethics of the Universitat Internacional de Catalunya. hMSCs from bone marrow were purchased from American Type Culture Collection. hMSCs were seeded in Dulbecco’s Modified Eagle’s Medium (DMEM) supplemented with 10% fetal bovine serum (FBS), 2 mM glutamax and 0.01% antibiotics and cultured on the incubator at 37 °C with 5% CO_2_ to a maximum confluence of 70%. For glucose deprivation experiments, the complete medium was substituted 3 days after seeding by DMEM without glucose supplemented with 1% FBS, 2 mM glutamax and 0.01% antibiotics either with or without 25 mM of glucose.

### Cell-lines

293 T cells (CRL3216, ATCC, Manassas VA, USA) derived from human embryonic kidney, SH-SY5Y (ATCC) derived from human neuroblastoma, and MCF-7 (Eucellbank, Barcelona, Spain) derived from human breast cancer.

### Lentiviral infection

Two lentiviral vectors, pWPI-IRES-GFP and pWPI-mCPT1C-IRES-GFP, were used to drive the expression of green fluorescence protein (GFP) and mouse CPT1C plus GFP, respectively^16^. Next, other two lentiviral vectors, pLVTHM-Random-IRES–GFP and pLVTHM-shCPT1C-IRES-GFP (random sequence or the previously validated silencing CPT1C sequences^13^), were constructed for human CPT1C silencing experiments.. The map and the sequences of these plasmids are available from Addgene (Cambridge, MA, UK). Lentiviruses were propagated and titrated as previously described^42^.

### Isolation of transduced cells

hMSCs were infected with 10 virus/cell and, 5 days later, separated by fluorescence emission (GFP) with a Fluorescence-Activated Cell Sorter (FACS).

### Real-time PCR

RNA was obtained using a TRIzol® kit (Invitrogen-Thermo Fisher) and template cDNA was prepared by reverse transcription. Gene expression was quantified by quantitative real-time PCR (qRT-PCR) using SYBR Green PCR Master Mix and the following primers: CPT1C *Forward*: 5′ GGACTGATGGAGAAGATCAAAGA 3′, CPT1C *Reverse*: 5′ CACAAACACGAGGCAAACAG 3′, β-actin *Forward*: 5′ CGTGATGGTGGGCATGGGTC 3′ and β-actin *Reverse*: 5′ ACGGCCAGAGGCGTACAGGG 3′ on a CFX96 real-time system (Bio-Rad). Relative gene expression between paired samples was estimated using the 2^-ΔΔCt^ method.

### Western Blot

Protein extracts were separated on SDS-polyacrylamide gels and transferred onto PVDF membranes. Blots were blocked and incubated at 4 °C overnight with primary antibodies. After washing, blots were incubated with horseradish peroxidase-conjugated secondary antibodies and developed using the LuminataTM Forte Western HRP substrate (Merck-Millipore, Darmstadt, Germany). Semiquantitative analysis was performed using densitometry with GeneTools software from SynGene (Cambridge, UK). The antibodies used were anti-mouse CPT1C antibody developed in our laboratory^8^, anti-rat CPT1A (LCPT I) antibody developed in France^43,44^, and the following commercially available antibodies: anti-human CPT1C purchased from Sigma (SAB2501194), which also recognizes mouse CPT1C (Supplemental Fig. 1C) due to the high similarity between the two species epitopes (93% of identity), anti-LC3A/B (#12741), and anti-GFP (#2956) from Cell Signaling Technology, anti-SQSTM1 from Abcam,, anti-β-actin from Thermo Fisher, and anti-GAPDH from Applied Biosystems.

### Immunocytochemistry

Mitotracker Orange CMTMRos (Thermo Fisher) was added to CPT1C-hMSCs at 500 nM and incubated for 30 minutes at 37 °C. Cells were washed, fixed with formaldehyde, permeabilized and blocked with 2% goat serum in PBS. Blocking solution was replaced by primary antibody solution: 1:30 of anti-mouse CPT1C produced in rabbits and anti-Climp63 mouse monoclonal antibody (Enzo Life Sciences, Lausen, Switzerland). After washing, cells were incubated with anti-rabbit AlexaFluor488 and anti-mouse AlexaFluor633 conjugated secondary antibodies (Thermo Fisher). Finally, coverslips were mounted. Imaging was performed on a confocal laser scanning ZEISS LSM 700 microscope (X63 objective, 8–12 z stacks, image bit depth 4–6 μm, resolution of 10.077 pixels per µm) For quantification, blinded sets of cells were cultured, stained simultaneously and imaged using identical settings. Quantitative imaging analyses were performed with the Imaris 8.5 processing package: surface-surface colocalization pluging was carried out in 3 independent experiments and 2 independent preparations, and at least 10 cells were analyzed for coverslip.

### Cell viability

Cell viability was analyzed by MTT assay in hMSCs cultured at 1% of serum to avoid proliferation of cells. After treatments, MTT solution was added at a final concentration of 0.2 mg/mL. The mitochondrial activity of live cells was measured by spectrophotometry at an absorbance wavelength of 570 nm.

### Measurement of ATP levels by bioluminescence

ATP determination kit (Thermo Fisher) was used. Cells were glucose deprived for 36 h, collected and processed as described by the manufacturer. Total cellular ATP levels were normalized to the total protein amount.

### Fatty acid oxidation (FAO) assay

Total palmitate oxidation was adapted from Roduit *et al*.^45^. Fatty acid oxidation to CO_2_ and acid-soluble products (ASP; essentially ketone bodies) were measured in hMSCs cultured in 25-cm^2^ flasks. Briefly, cells were washed in KRBH-0.1% BSA, pre-incubated for 30 minutes in KRBH-1% BSA, and then incubated for 3 h with fresh KRBH containing 8 mM carnitine, and 300 µM [1-^14^C] palmitate-BSA (GE Healthcare, Little Chalfont, UK) in the presence or absence of 5 mM glucose in a CO_2_-free incubator. The flasks were sealed with a stopper supporting a 3-cm length of PVC tubing containing a piece of Whatman GF/B paper soaked in 0.1 N KOH. At the end of the incubation period, 0.2 mL of perchloric acid was injected into each flask to acidify the medium and liberate the CO_2_. After overnight isotopic equilibration, the trapped ^14^CO_2_ was measured by liquid scintillation counting. FAO into ASP was measured from the perchloric acid–treated medium after centrifugation. Next, supernatants containing the labeled ASP were collected and counted by liquid scintillation. The scintillation values were normalized to the protein content.

### Lipid droplet (LD) staining

Neutral lipids were stained in fixed cells with Nile Red or Bodipy (Sigma-Aldrich, Saint Louis, MO, USA) to a final concentration of 0.5 µg/mL and cell nuclei were stained with Hoechst. Imaging was performed with a semi-confocal Nikon Ti microscope (X63 objective) with an Andor DSD camera. Measurements of randomly selected cells were made with the Fiji image processing package.

### TAG lelvels

Total triglyceride content was determined using the TAG determination kit according to the manufacturer (Sigma-Aldrich, Saint Louis, MO, USA). TAG values were normalized to the protein content. TAG content.

### Statistics

Statistical analysis was performed using PRISM (GraphPad Software). According to data normality (Shapiro-Wilk test), significance between 2 groups was determined using either the Student’s *t-*test or a Mann-Whitney U test (parametric and non-parametric, respectively). For comparisons among 3 to 4 groups, the ANOVA test was performed, followed by the Bonferroni post-test.

### Data availability

All data generated or analyzed during this study are included in this published article (and its Supplementary Information files).

## Electronic supplementary material

Supplementary information
