# Supplementary material for: CPT1C promotes human mesenchymal stem cells survival under glucose deprivation through the modulation of autophagy
Source: Sci Rep. 2018 May 3;8:6997. doi: 10.1038/s41598-018-25485-7 (PMC5934389; doi:10.1038/s41598-018-25485-7)
Supplement: Supplementary file 1 — Supplementary information [file 41598_2018_25485_MOESM1_ESM.pdf]

Supplemental information about:

CP1C promotes human mesenchymal stem cells survival under glucose deprivation through the modulation of autophagy

Xavier Roa-Mansergas[1,*], Rut Fadó[1,*], Maher Atari[2], Joan F Mir[3], Helena Muley[1], Dolors Serra[3,4], Núria Casals[1,4,#]

Supplemental methods

Cell mortality. The percentage of cell mortality was analyzed by trypan blue exclusion. Cultures were washed and collected by tripsinization. Then, cells were stained with 0.4% of trypan blue (Sigma-Aldrich, Madrid, Spain). The number of live (unstained) and dead (blue) cells were counted using a microscope.

Bone marrow hMSC and MCF7 cells. Bone marrow-derived MSCs were purchased from ATCC at 2 passages and breast cancer cell line MCF7 was obtained from Eucellbank at 12 passages (University of Barcelona, Barcelona, Spain), expanded in supplemented DMEM medium and cultured in the incubator at 37ºC with 5% CO_2 to a maximum confluence of 80%. To achieve a stable infection, BM-hMSCs were infected and separated by FACS as described in the hMSC dental pulp. Bone marrow-derived MSCs at 10-12 passages were used for survival experiments.

Osteogenic and adipogenic differentiation of hMSCs. Control cells, CPT1C-hMSCs, and EV-hMSCs at 14 passages were used. For osteogenic differentiation, cells were seeded at 25,000 cells/ml in osteogenic medium: RPMI Glutamax Medium (Gibco-Thermo Fisher, Waltham, MA, USA), 10% of FBS, 20 mM of β-glycerol phosphate, 50 µM of L-ascorbic acid, 10 nM of dexamethasone, and 1% of antibiotic. For adipogenic differentiation, cells were seeded at 125,000 cells/ml in adipogenic medium: DMEM medium, 10% of FBS, 0.5mM of isobutylmethylxanthine, 10µM of insulin, 200µM of indomethacin, 1µM of dexamethasone, and 1% of antibiotic. Cells were maintained in the incubator at 37ºC with 5% CO_2, with the medium being replaced every 3 to 4 days until 21 days had passed.

Alizarin red stain. hMSC osteogenic differentiated cells were washed twice with PBS and fixed with 10% of formalin for 15 minutes at 4ºC. After 2 washes, 2% of Alizarin Red diluted in water at a pH of 4.2 was added and incubated for 45 minutes at room temperature. Stained cells were washed with water several times. hMSCs, CPT1C-hMSCs, and EV-MSC undifferentiated cells seeded 3 days before staining were used as negative controls.

Oil red stain. hMSC adipogenic differentiated cells were washed twice with PBS and fixed. After several washes with H_2O, 0.5% of Oil Red solution in 60% of isopropanol was added and cells incubated for 15 minutes at room temperature. Stained cells were washed with water several times.

Osteogenic and adipogenic marker detection by real-time PCR. For expression analysis, template cDNA was amplified using the following primers: Collagen 1 *Forward*: 5’ CAG TCT GCT GGT CCA TGT A 3’, Collagen 1 *Reverse*: 5’ ACT GGT GAG ACC TGC GTG TA 3’, CEBPα *Forward*: 5’ CAC AGC GAC GAC TGC

AAG ATC C 3’ and CEBPα *Reverse*: 5’ CTT GAA CAA GTT CCG CAG GGT G 3’ in a real-time system. hMSC, CPT1C-hMSC and EV-hMSC undifferentiated cells were used as negative controls.

Human CPT1C and p62 detection by real-time PCR. For expression analysis, template cDNA was amplified using the following primers: Human CPT1C *Forward*: 5' GGA CTG ATG GAG AAG ATC AAA GA 3’, Human CPT1C *Reverse*: 5’ CAC AAA CAC GAC GCA AAC AG 3’, p62 *Forward*: 5’ CTG GGA CTG AGA AGG CTC AC 3’ and p62 *Reverse*: 5’ GCA GCT GAT GGT TTG GAA AT 3’ in a real-time system.

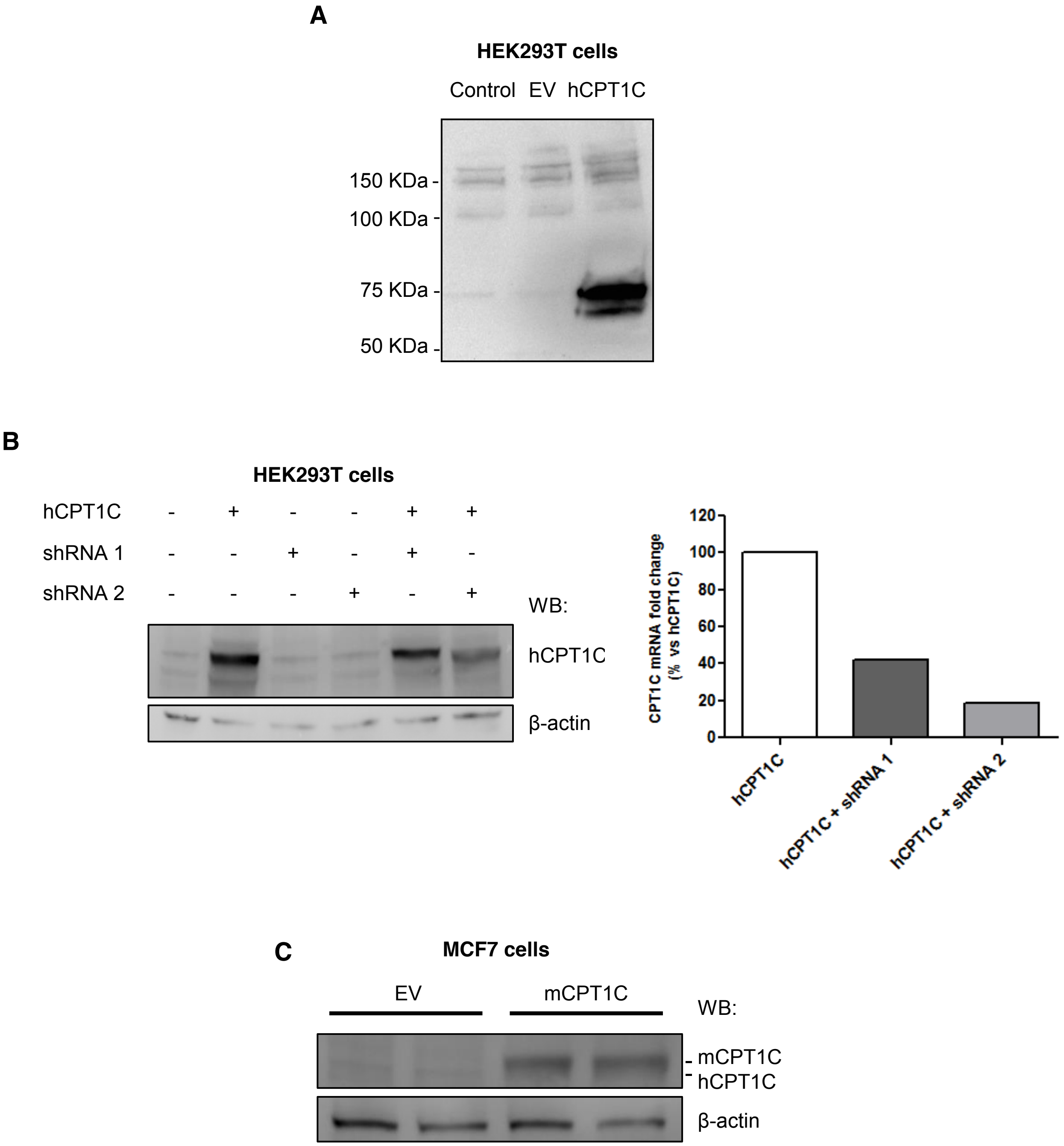

Supplemental Figure 1. Human CPT1C detection and silencing. (A) HEK293T cells were transfected with an empty vector (EV, pIRES2-EGFP vector from Clontech, BD Biosciences) or a vector expressing human CPT1C (pIRES2-hCPT1C-EGFP). Non-transfected HEK293T cells were used as a control. An intense 75-kDa band was found in hCPT1C overexpressing cells. (B) Then, cells were cotransfected with two different silencing sequences for human CPT1C (shRNA1 or shRNA 2) subcloned in the pLVTHM-IRES-GFP vector. As shown in the graph, shRNA number 2 exerted a stronger effect in reducing CPT1C protein levels. Here forward, only this sequence was used and called sh hCPT1C. (C) Breast cancer cell line MCF7 was transduced with lentivirus containing the empty vector (EV) or the vector codifying for mouse isoform of CPT1C and subsequently separated by FACS. Endogenous human CPT1C and overexpressed mouse isoform were detected by western blot using a commercially available anti-CPT1C antibody produced with a human peptide. It is observed that human CPT1C presents a molecular weight slightly lower than mouse form. β-actin was used as a loading control.

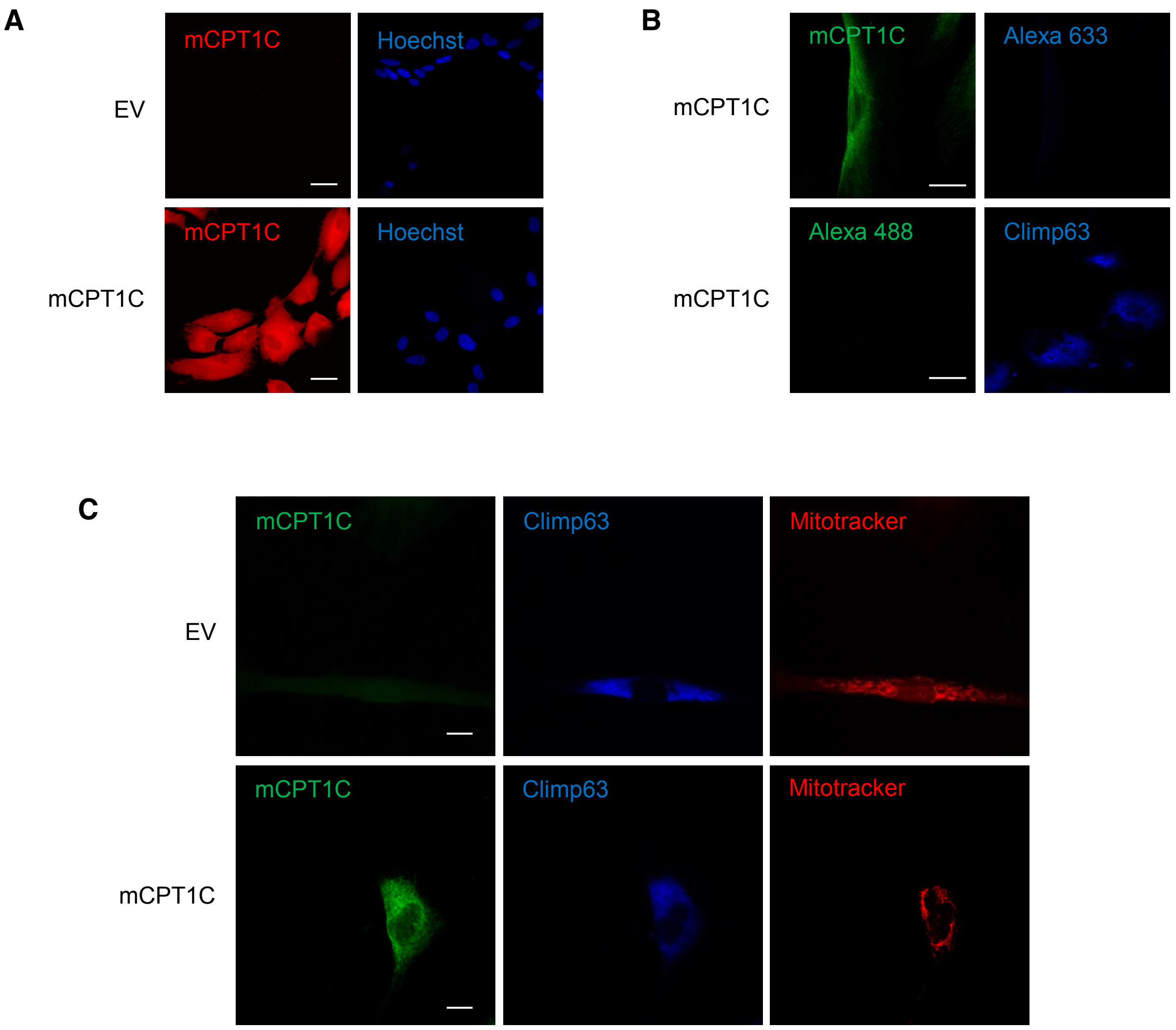

Supplemental Figure 2. Anti-mCPT1C antibody specificity in MSCs cells. (A) Primary antibody specificity. hMSCs were infected with a lentivirus to drive the expression of mouse CPT1C (pWPI-mCPT1C-IRES-GFP). Anti-mCPT1C antibody and a goat anti-rabbit Alexa Fluor 568 conjugated antibody was used to detect mouse CPT1C by immunocytochemistry. EV-hMSCs were used to demonstrate primary antibody specificity. Scale bar 20µm. (B) Secondary antibodies specificity. Anti-mCPT1C antibody and goat anti-rabbit Alexa Fluor 488 conjugated antibody were used to detect CPT1C, while anti-Climp63 and goat anti-mouse Alexa Fluor 633 conjugated antibody were used to stain the ER. In the upper sample, the primary antibody against the ER marker was not added, while in the lower one, the antibody against CPT1C was absent. Scale bar 20µm. (C) Immunochemistry of mCPT1C in control cells. EV and CPT1C-hMSCs were stained as in Figure 1E. Scale bar 10µm. Images of EV-hMSCs demonstrate that the GFP signal was negligible in those acquisition conditions.

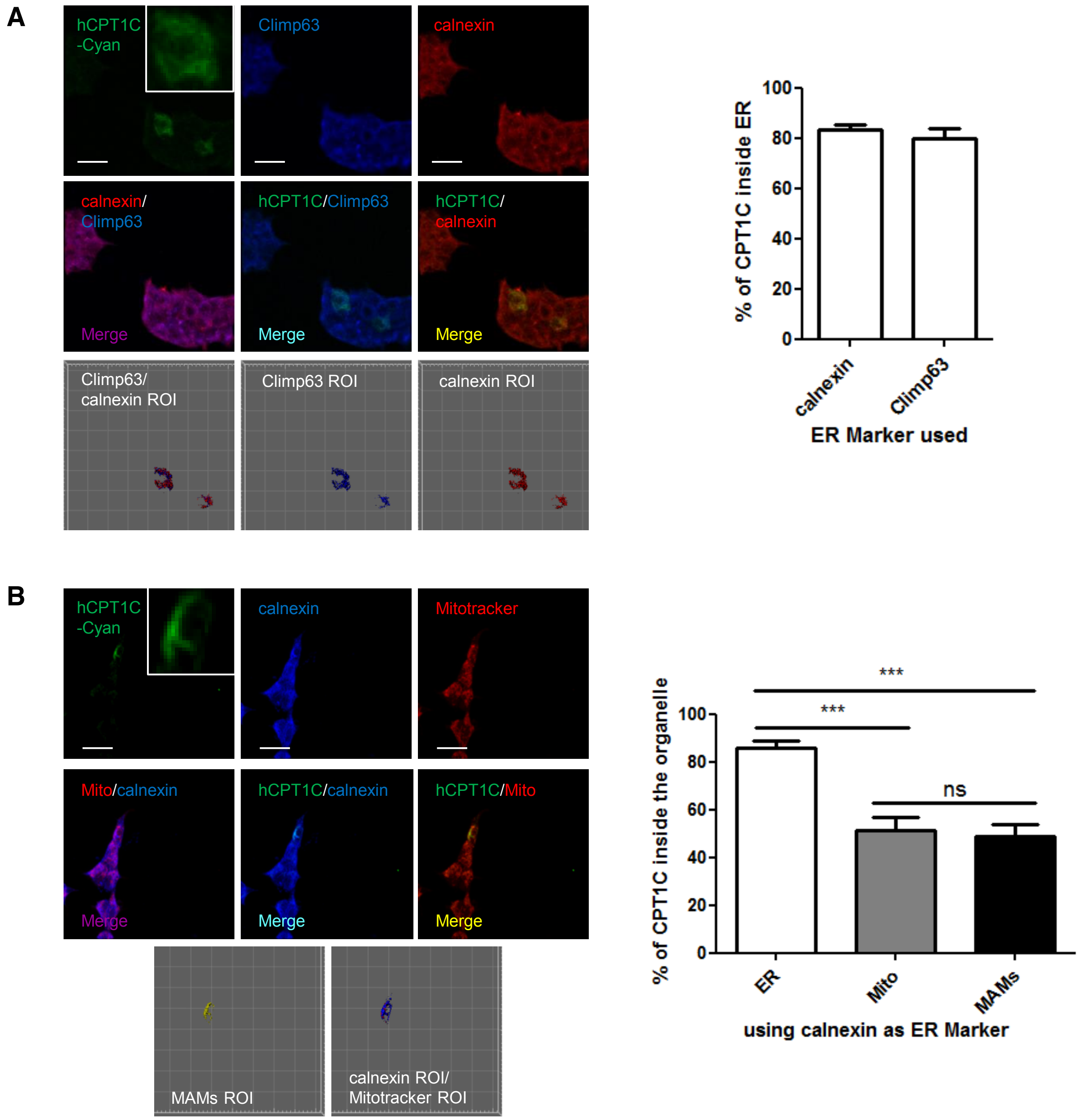

Supplemental Figure3. Human CPT1C localization in HEK293T cells. Cells were transfected with pmCPT1C-turquoise (empty vector obtained from Addgene) and fixed 24 hours later. Climp63 and calnexin were used as ER markers and detected by immunocytochemistry. Mitotracker Orange was used to stain mitochondria. The graph in A shows the percentage of CPT1C inside the ER using calnexin or Climp63 as ER marker and in B the percentage of CPT1C inside the ER, in the mitochondria, or on the surface defined by the colocalization of the ER with the mitochondria (MAMs) using calnexin as ER marker. Values are expressed as mean ± SEM of ten cells in each experiment. Scale bar 30μm. ****p* <0.001.

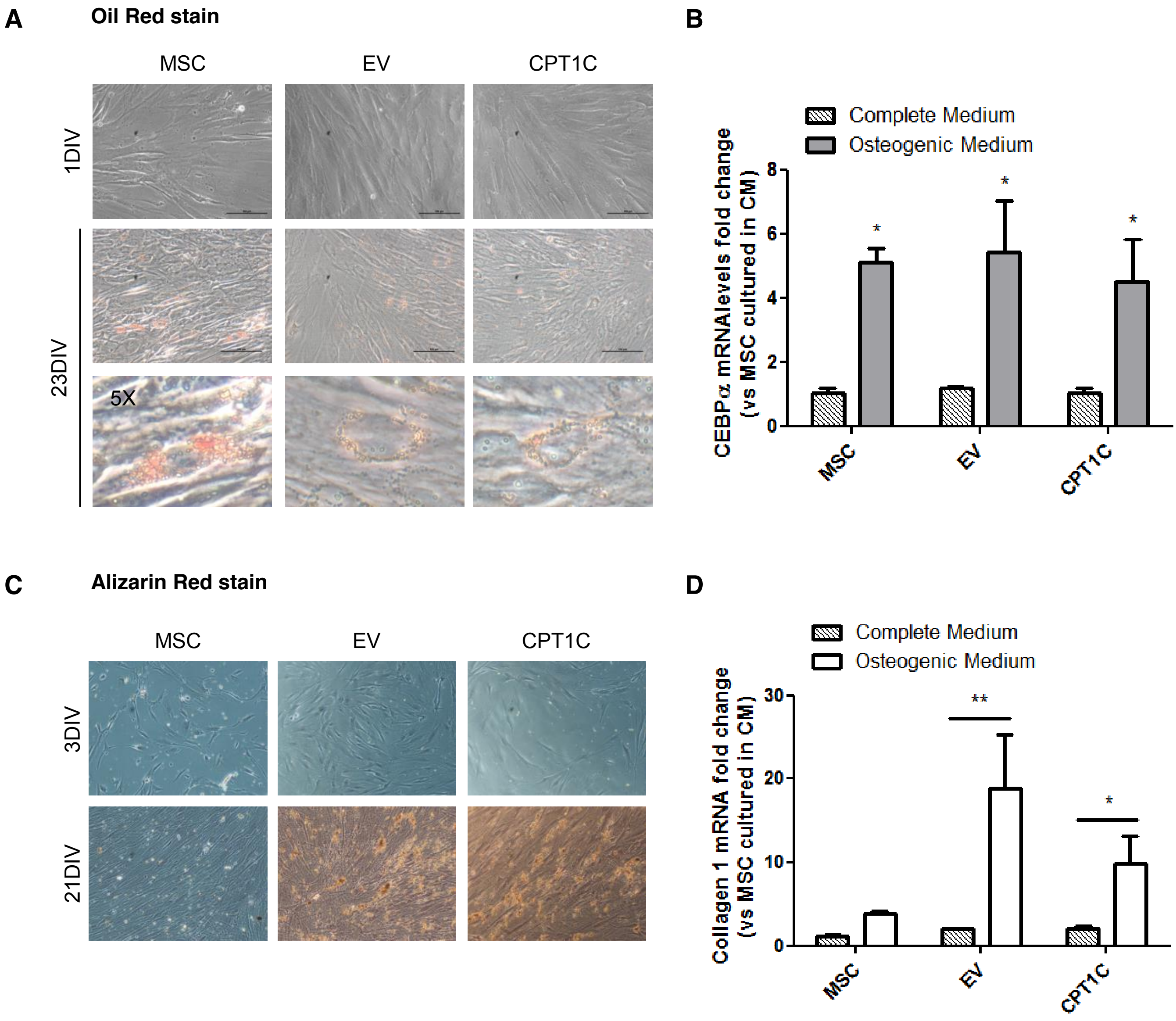

Supplemental Figure 4. Adipogenic and osteogenic differentiation. (A) hMSCs, EV-hMSCs, and CPT1C-hMSCs at 14 passages were cultured in adipogenic medium for 23 days. Next, Oil Red staining was performed to confirm lipid deposition. (B) CEBPα mRNA expression was detected as an adipogenic marker (B). The graph shows the mean ± SEM of quadruplicates. Scale bar 100µm. (C) hMSCs, EV-hMSCs, and CPT1C-hMSCs at 14 passages were cultured in osteogenic medium for 21 days. Next, Alizarin Red staining was performed to confirm calcium deposition. (D) Collagen 1 mRNA expression was detected as an osteogenic marker. Uninfected hMSCs were used as positive controls. Cells seeded in complete medium for 3 days were used as negative controls. The graph shows the mean ± SEM of the quadruplicates. **p* <0.05; ****p* <0.001.

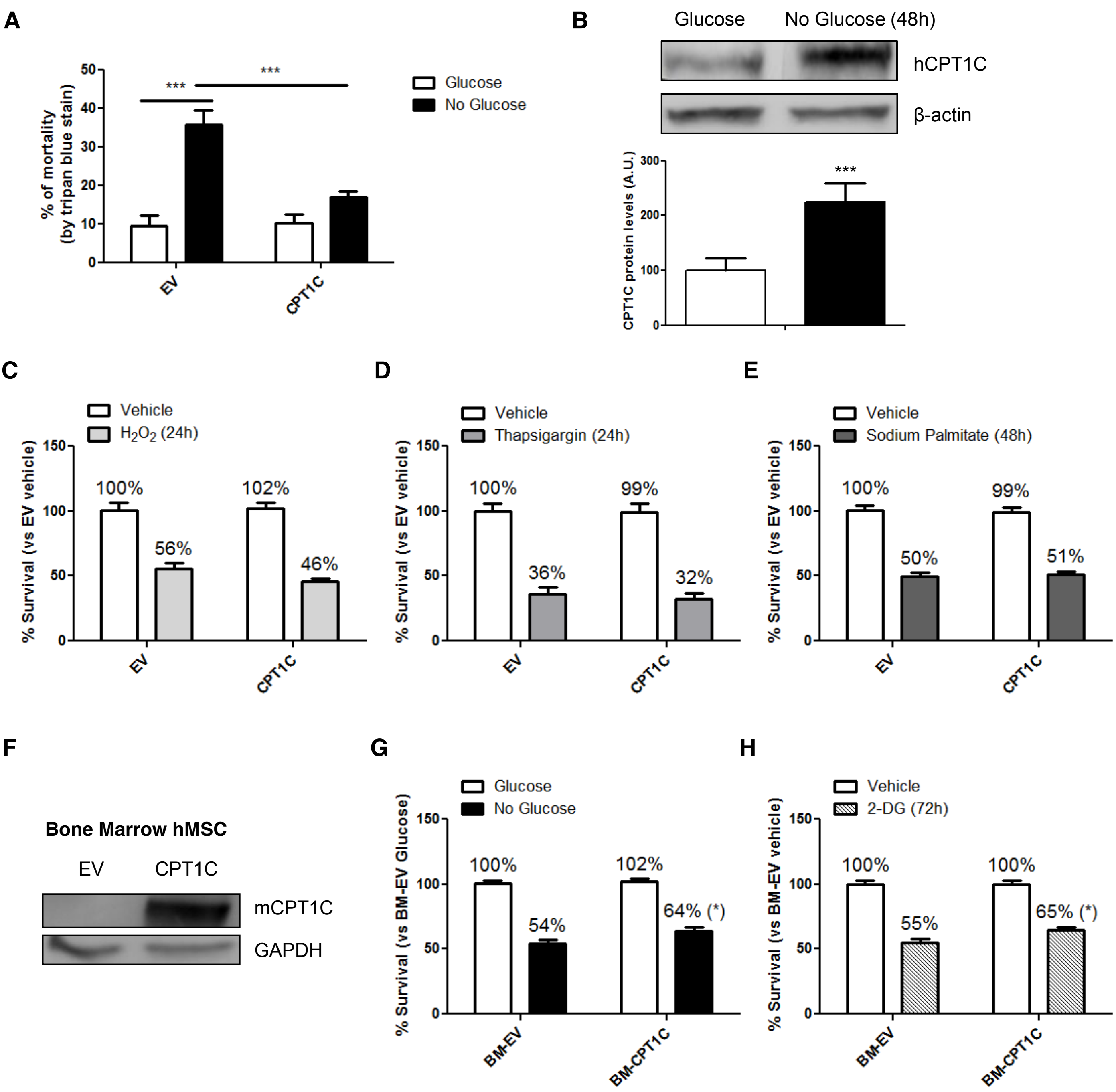

Supplemental Figure 5. Cell survival analysis under glucose deprivation, oxidative stress, lipotoxicity, or endoplasmic reticulum stress. (A) EV or CPT1C-hMSCs were glucose deprived for 24 h. The percentage of cellular mortality was analyzed by tripan blue stain. (B) Endogenous protein levels of CPT1C in hMSC were analyzed ny WB at 48 hours after glucose deprivation. Graph shows the mean ± SEM of 4 independent experiments. (C-E) EV or CPT1C-hMSCs were treated with 150 μM H_2O_2 for 24 h (C), 10 μM thapsigargin for 24 h (D), or 500 μM sodium palmitate for 48 h (E) and cellular survival was analyzed by MTT reduction assay. (F-H) hMSCs from bone marrow were transduced with pWPI-IRES-GFP (EV) or pWPI-mCPT1C-IRES-GFP (CPT1C) and selected by FACS. Mouse CPT1C overexpression in selected cells was assessed by Western Blot (F). Cell viability (MTT assay) was assessed in EV or CPT1C bone marrow hMSCs after glucose deprivation (FG or 2-DG treatment (H). All results are shown as mean ± SEM of 3 independent experiments performed in sextuplet. **p* <0.05; ***p* <0.01; ****p* <0.001

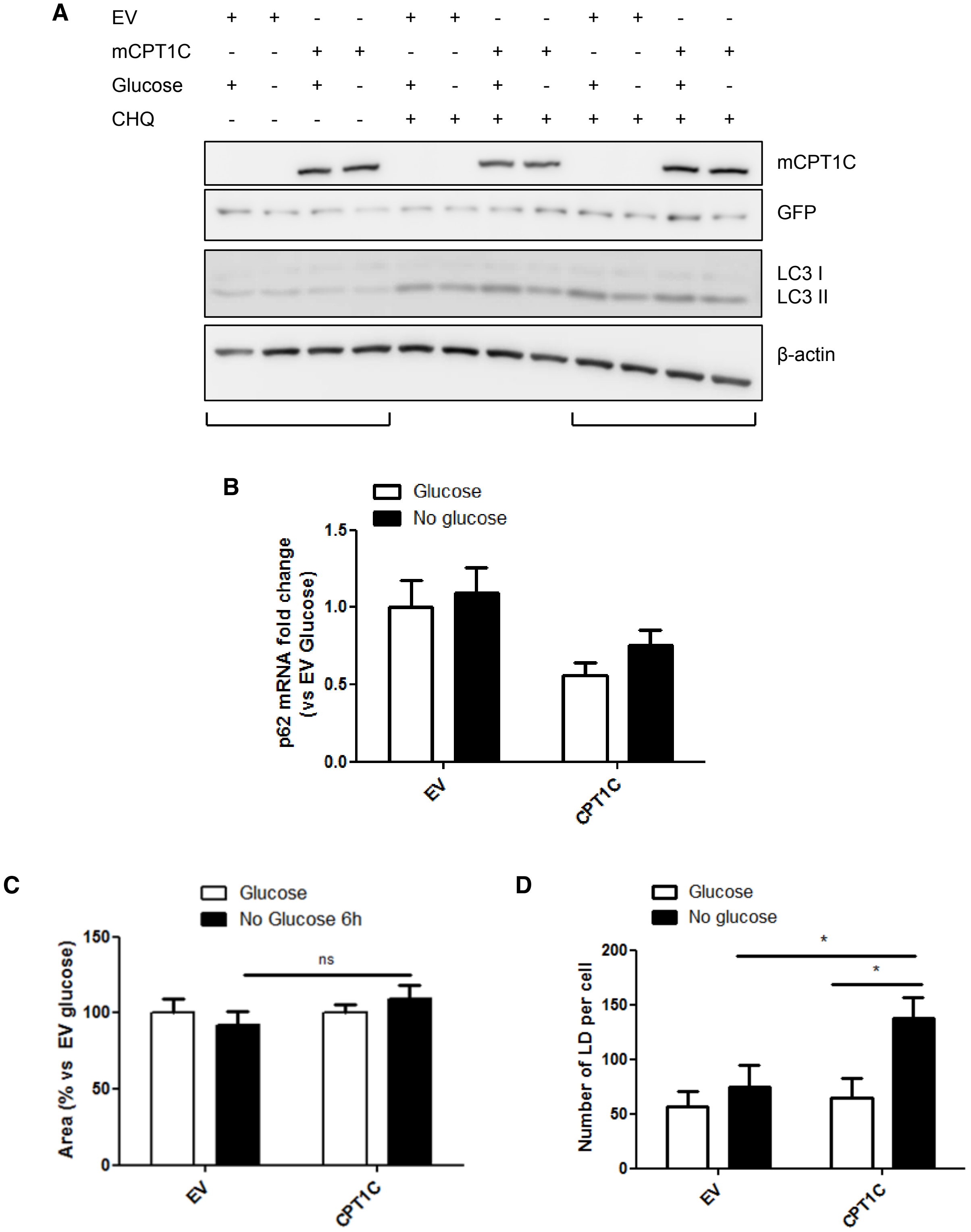

Supplemental Figure 6. CPT1C overexpression promotes autophagy and LD synthesis under glucose-depleted conditions. (A) Full-length blot from Figure 4A. LC3 I and LC3 II were analyzed by WB in EV and CPT1C-hMSCs in the presence or the absence (24 h) of glucose. GFP was used as a transduction control and β-actin as a loading control. Bars below indicate the cropped regions shown in Figure 4A. (B) p62 mRNA level were analyzed by RT-PCR in EV or CPT1C-MSCs 24 hours after glucose starvation. Graph shows the mean ± SEM of 2 independent experiments performed in triplicate. (C) Mean area of LDs from Figure 6B. LDs were stained with Nile Red. Results are shown as the mean ± SEM of 3 independent experiments performed in triplicate. (D) Analysis of LD number by Bodipy staining in EV or CPT1C-hMSCs cultures in the presence or the absence (6h) of glucose. Graph shows the mean ± SEM of 10 cells in each condition. *$p < 0.05$

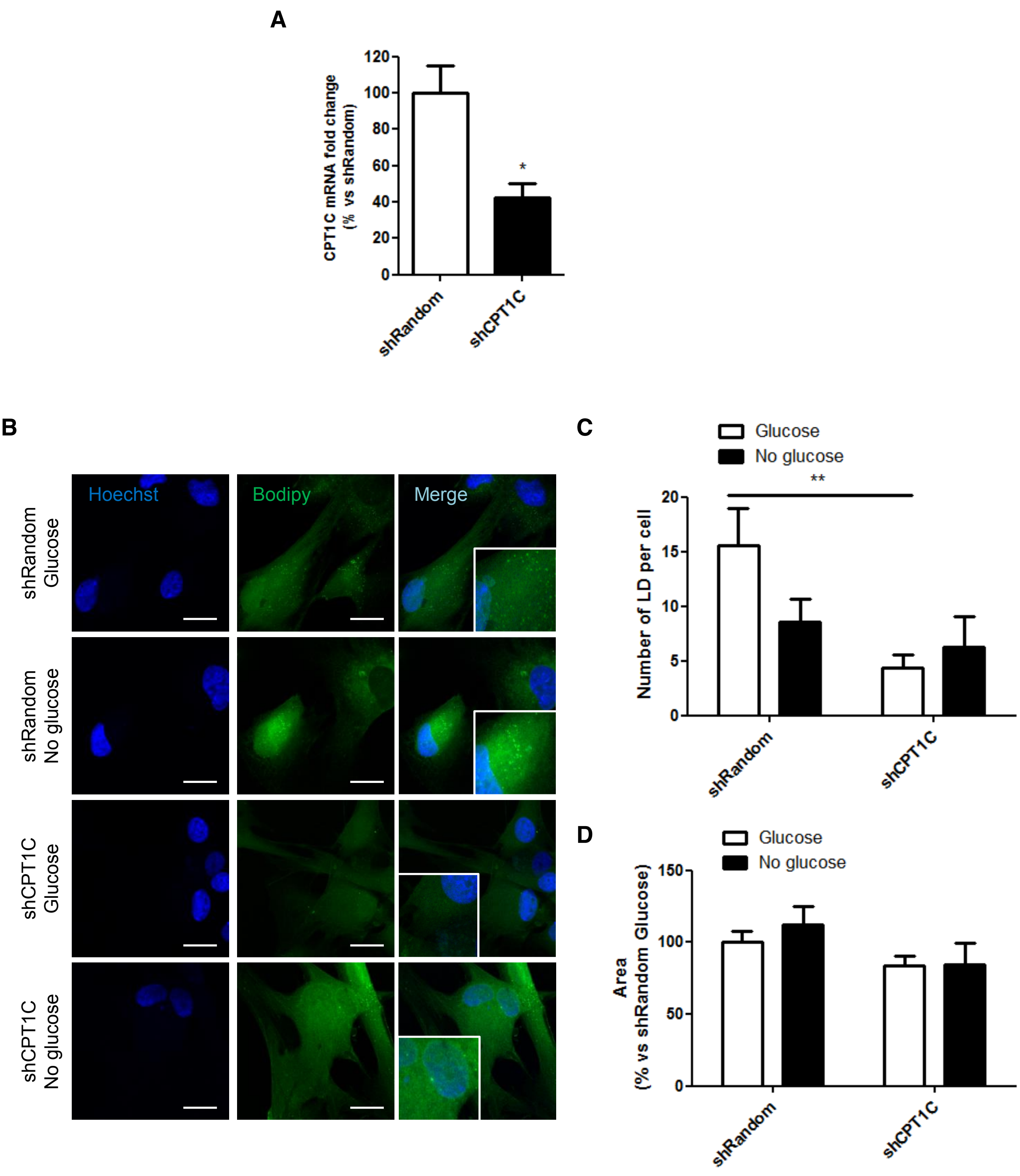

Supplemental Figure 7. Deregulation of LD number in CPT1C deficient MSCs. (A) CPT1C downregulation was corroborated at mRNA level by RT-PCR in shCPT1C-MSCs. Graph shows the mean ± SEM of quadruplicates. (B-D) Analysis of LD number (C) and area (D) in shRandom or shCPT1C-hMSCs cultured with or without glucose for 6 h. Cells were stained with Hoechst (blue) or Bodipy (green). Representative images are shown in B. The number and the area of LDs per cell are shown as the mean ± SEM of 2 independent experiments performed in duplicate. 50 cells were analyzed in each condition. Scale bar 20 μm. **p <0.01
